# The Hemiparasitic Plant *Phtheirospermum* (Orobanchaceae) Is Polyphyletic and Contains Cryptic Species in the Hengduan Mountains of Southwest China

**DOI:** 10.3389/fpls.2018.00142

**Published:** 2018-02-09

**Authors:** Wen-Bin Yu, Christopher P. Randle, Lu Lu, Hong Wang, Jun-Bo Yang, Claude W. dePamphilis, Richard T. Corlett, De-Zhu Li

**Affiliations:** ^1^Center for Integrative Conservation, Xishuangbanna Tropical Botanical Garden (CAS), Mengla, China; ^2^Southeast Asia Biodiversity Research Institute, Chinese Academy of Sciences, Yezin, Myanmar; ^3^Department of Biological Sciences, Sam Houston State University, Huntsville, TX, United States; ^4^Key Laboratory for Plant Diversity and Biogeography of East Asia, Kunming Institute of Botany, Chinese Academy of Sciences, Kunming, China; ^5^Plant Germplasm and Genomics Center, Germplasm Bank of Wild Species, Kunming Institute of Botany, Chinese Academy of Sciences, Kunming, China; ^6^Department of Biology, Graduate Program in Plant Biology, The Pennsylvania State University, State College, PA, United States

**Keywords:** cryptic species, Hengduan Mountains, Orobanchaceae, *Phtheirospermum*, phylogenetic incongruence, *Pterygiella*

## Abstract

*Phtheirospermum* (Orobanchaceae), a hemiparasitic genus of Eastern Asia, is characterized by having long and viscous glandular hairs on stems and leaves. Despite this unifying character, previous phylogenetic analyses indicate that *Phtheirospermum* is polyphyletic, with *Phtheirospermum japonicum* allied with tribe Pedicularideae and members of the *Ph. tenuisectum* complex allied with members of tribe Rhinantheae. However, no analyses to date have included broad phylogenetic sampling necessary to test the monophyly of *Phtheirospermum* species, and to place these species into the existing subfamiliar taxonomic organization of Orobanchaceae. Two other genera of uncertain phylogenetic placement are *Brandisia* and *Pterygiella*, also both of Eastern Asia. In this study, broadly sampled phylogenetic analyses of nrITS and plastid DNA revealed hard incongruence between these datasets in the placement of *Brandisia*. However, both nrITS and the plastid datasets supported the placement of *Ph. japonicum* within tribe Pedicularideae, and a separate clade consisting of the *Ph. tenuisectum* complex and a monophyletic *Pterygiella*. Analyses were largely in agreement that *Pterygiella*, the *Ptheirospermum* complex, and *Xizangia* form a clade not nested within any of the monophyletic tribes of Orobanchaceae recognized to date. *Ph. japonicum*, a model species for parasitic plant research, is widely distributed in Eastern Asia. Despite this broad distribution, both nrITS and plastid DNA regions from a wide sampling of this species showed high genetic identity, suggesting that the wide species range is likely due to a recent population expansion. The *Ph. tenuisectum* complex is mainly distributed in the Hengduan Mountains region. Two cryptic species were identified by both phylogenetic analyses and morphological characters. Relationships among species of the *Ph. tenuisectum* complex and *Pterygiella* remain uncertain. Estimated divergence ages of the *Ph. tenuisectum* complex corresponding to the last two uplifts of the Qinghai–Tibet Plateau at around 8.0–7.0 Mya and 3.6–1.5 Mya indicated that the development of a hot-dry valley climate during these uplifts may have driven species diversification in the *Ph. tenuisectum* complex.

## Introduction

Mountain ranges often support a high diversity of plant life (Favre et al., 2015; Hughes and Atchison, 2015). Many of the 35 biodiversity hotspots are located in mountain ranges (Myers et al., 2000; Mittermeier et al., 2011). The Hengduan Mountains region is the core of the Mountains of Southwest China Biodiversity Hotspot (Zhang et al., 2009; Boufford, 2014). This region is characterized by extremely complex topography, with altitudes ranging from less than 2,000 meters in some valley floors to 7,558 meters at the summit of Gongga Mountain. Generally, the mountain ridges are oriented in the north–south direction, perpendicular to the main Himalayas. Several major river systems originated or were reorganized in this region during the uplift of the mountains, e.g., the Jinshajiang (Upper Yangtze) and its tributaries (Yalongjiang, Daduhe, Jialingjiang), as well as the Salween (Nujiang), and Mekong (Lancangjiang) (Zhang et al., 2011c). The Hengduan Mountains region harbors more than 9,000 species of vascular plants, around 32% of which are endemic (Li and Li, 1993; Wang and Wu, 1993, 1994; Zhang et al., 2009). More than 30 genera are endemic to this region and the adjacent Himalayas (Ying and Zhang, 1984; Wu et al., 2005; Boufford, 2014). Of these, several endemic genera have been subsumed within other widely distributed genera (Friesen et al., 2000; Pan et al., 2009; Tian et al., 2011; Zhang et al., 2011b; Nie et al., 2013). However, discovery of new genera is ongoing (Al-Shehbaz et al., 2004; Zhang et al., 2011a; Wang W. et al., 2013; Wang Y.-J. et al., 2013). Species richness and diversity in the Hengduan Mountains region have been ascribed to the accumulation of migrants and *in situ* diversification accelerated by the uplift of the mountains (Wen et al., 2014; Xing and Ree, 2017). This uplift event may have contributed to recent diversification of species in *Phtheirospermum* Bunge (Orobanchaceae), most of which can be found in the Hengduan Mountains region.

*Phtheirospermum* is a genus of hemiparasitic plants characterized by having long and viscous glandular hairs on stems and leaves, yet monophyly and species boundaries remain uncertain. Historically, this genus was divided into five species. Tao (1996) organized species in two sections in a full revision of the genus; section *Phtheirospermum* contains *Ph. japonicum* (Thunb.) Kanitz and *Ph. tenuisectum* Bureau & Franch. The former is an experimental model for the study of genetics and development of the haustorium in a generalist parasite (Cui et al., 2016; Ishida et al., 2016). Section *Minutusepala* (not validly published) includes the remaining species, *Ph. glandulosum* (Benth.) Hook. f., *Ph. muliense* C. Y. Wu & D. D. Tao and *Ph. parishii* Hook. f. Based on the Chinese material of *Ph. glandulosum*, Hong (1979) established *Pseudobartsia*, with one species *Pseudobartsia yunnanensis* Hong (Tao, 1993); because *Ps. yunnanensis* cannot be distinguished from *Ph. glandulosum*, Yu et al. (2015b) created the combination *Ps. glandulosum*. Molecular and morphological evidence supports the independent generic status of *Pseudobartsia* (Lu et al., 2007; Dong et al., 2013, 2015).

Of the remaining four species of *Phtheirospermum*, *Ph. japonicum* occurs throughout China (except Xinjiang), extending to eastern Russia, the Korean Peninsula, and Japan; *Ph. tenuisectum* is restricted to Western China (Guizhou, Qinghai, Sichuan, and Xizang); *Ph. muliense* is known by only the type collection from Muli in Sichuan, southwestern China; and *Ph. parishii* occurs in southern Myanmar and Thailand (Yamazaki, 1990). Tao (1996) reported three collections for *Ph. parishii* in Sichuan. Based on comparisons of type materials and herbarium specimens, however, the three gatherings, including seven sheets conserved at CDBI, KUN, and PE, are short plants of *Ph. tenuisectum*, and all of them were originally identified as *Ph. tenuisectum*. *Phtheirospermum parishii* was firstly collected from Tanintharyi region of southern Myanmar, then it was also found in northern Thailand (Yamazaki, 1990).

Even with *Ph. glandulosum* excluded from *Phtheirospermum*, monophyly of the remaining species is still not resolved. Phylogenetic analyses including *Ph. japonicum* or *Ph. tenuisectum* alone indicates that *Ph. japonicum* is close to tribe Pedicularideae (Bennett and Mathews, 2006; McNeal et al., 2013), whereas *Ph. tenuisectum* was excluded from tribe Pedicularideae as an independent lineage (Ree, 2005). Dong et al. (2013) were the first to sample three species of *Phtheirospermum*, *Ph. japonicum*, *Ph. tenuisectum*, and *Ph. muliense*. However, they included the sample *J. S. Ying 4144* from Sichuan, which is one of three gatherings misidentified as *Ph. parishii* by Tao (1996). The phylogenetic analyses showed that *Phtheirospermum* spp. separated into two clades: one clade including *Ph. japonicum* as sister to *Pedicularis* spp., and another clade includes *Ph. muliense* and *Ph. tenuisectum*, as sister to *Pterygiella*. In the study of Yu et al. (2015a), *Ph. tenuisectum* and *Ph. japonicum* were included as outgroups; similar to previous studies, *Ph. tenuisectum* is resolved as sister to *Pterygiella nigrescens*, while *Ph. japonicum* is included in a clade of tribe Pedicularideae. Accompanied by a broad sampling of tribe Rhinantheae *sensu lato*, Pinto-Carrasco et al. (2017) used the data of the “*Pterygiella* complex” from Dong et al. (2013) to recover the polyphyly of *Phtheirospermum*. Although polyphyly of *Phtheirospermum* is suggested by these prior studies, complete species sampling and placement of species in a broad phylogenetic context of the family Orobanchaceae are needed. In the more broadly sampled study of McNeal et al. (2013), *Pterygiella* was placed at the base of tribe Rhinantheae *sensu stricto* using *PHYA* and *PHYB* datasets, but was weakly supported as sister to *Brandisia* using nrITS and the plastid datasets. In this study, *Brandisia* was close to tribe Cymbarieae, or sister to the clade including tribes Pedicularideae + Buchnereae + Rhinantheae (including *Pterygiella*) using *PHYA* and *PHYB* datasets, respectively. Therefore, instability of the placement of *Brandisia* and *Pterygiella* casts further into doubt the placement of *Ph. tenuisectum* and *Ph. muliense*, and fails to provide an indication as to which, if any, existing tribes these genera can be placed.

**FIGURE 1:**
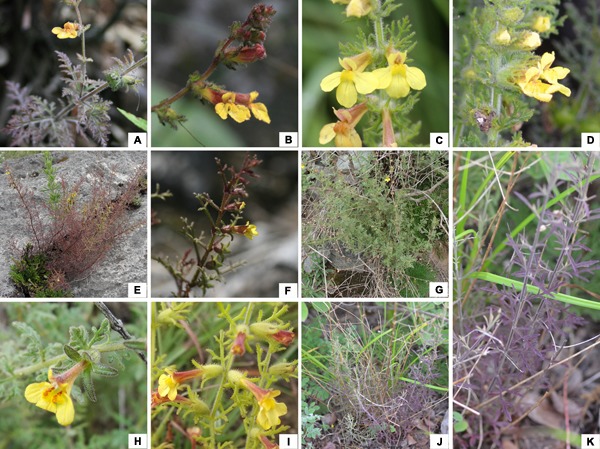
Plants of the *Phtheirospermum tenuisectum* complex. **(A–D)** Morphological variation in *Ph. tenuisectum*: **(A)** dissected leaves with wide lobes, and **(B)** flowers with spreading lower-lip and entire calyx lobes from Kangding (population T6, see **Figure 2** and **Supplementary Table S1**, hereafter); **(C)** yellow flower with drooping lower-lip and branched calyx lobes from Lijiang (T12); and **(D)** yellow flower with spreading lower-lip and branched calyx lobes (T2). **(E,F)** *Phtheirospermum* sp. 1 from Lijiang (D1 and D2): **(E)** a whole plant and habitat; **(F)** flowering branch. **(G–I)** Morphological variation in *Phtheirospermum* sp. 2: **(G)** a flowering plant, and **(H)** leaves/bracts and flower with long and entire calyx lobes from Batang (H7); and **(I)** leaves with linear lobes from Zhongdian (H2). **(J,K)** *Ph. muliense*: **(J)** plants and habitat; **(K)** vegetative branch.

Currently, two species of *Phtheirospermum*, *Ph. japonicum* and *Ph. tenuisectum*, have been adopted in Chinese Floras (Tsoong and Yang, 1979; Hong et al., 1998). Morphologically, it is easy to distinguish *Ph. tenuisectum* from *Ph. japonicum* in that the former has dissected leaves with linear pinnae (vs. narrowly ovate to orbicular pinnae in the latter), smaller yellow corollas (vs. red/pink corollas), and smaller fruits and seeds. In herbaria, specimens having dissected leaves with linear pinnae, yellow flowers and small fruits, have almost all been labeled as *Ph. tenuisectum*. Based on examination of herbarium specimens, we found that *Ph. tenuisectum* varied extensively in habit, leaf morphology, calyx form, and corolla shape. With further observation of fresh materials in the field, we have recognized four distinct morphotypes (**Figure 1**). These four morphotypes appear to be of the same lineage, called the *Ph. tenuisectum* complex hereafter. One morphotype with dimorphic leaves (**Figures 1J,K**) has been described as *Ph. muliense* (Tao, 1996), which has been supported by molecular analyses (Dong et al., 2013). Except for *Ph. tenuisectum* (**Figures 1A–D**), the remaining two morphotypes are treated as two new taxa (species 1: **Figures 1E,F**; species 2: **Figures 1G–I**).

The main goals of this study were to: (1) investigate the suspected polyphyly of *Phtheirospermum*; (2) delimit morphology-based species using molecular and phylogenetic approaches, and (3) estimate the evolutionary histories of species in *Phtheirospermum*. To achieve our goals, we sampled the *Ph. tenuisectum* complex extensively. A population of *Ph. muliense* was newly discovered in Shangri-La, in northwestern Yunnan. Two individuals from this population were sequenced in this study. We reconstruct the phylogeny of the *Ph. tenuisectum* complex, and re-evaluated the phylogenetic relationship between the *Ph. tenuisectum* complex and *Ph. japonicum* on the base of large-scale sampling of *Phtheirospermum*, as well as selected genera from six recognized tribes (McNeal et al., 2013), and *Brandisia*, and *Rehmannia* (Stevens, 2001 onward; Xia et al., 2009; Zeng et al., 2017). To understand the evolutionary history of *Phtheirospermum*, divergence times were estimated using large-scale sampling of Lamiales and four fossil calibrations.

## Materials and Methods

### Taxon Sampling

**FIGURE 2:**
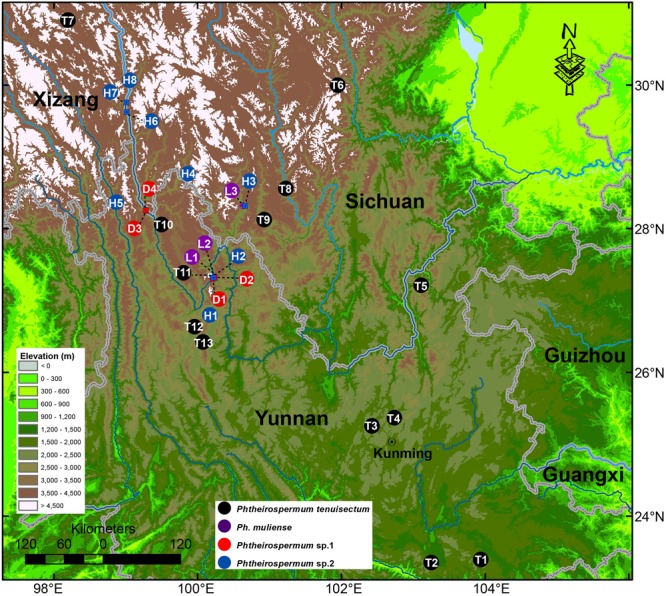
Geographical sampling of the *Ph. tenuisectum* complex in Southwest China. The river systems are highlighted in blue. The color of circles corresponds to taxa. Numbers and letters indicate populations; these abbreviations are also used in the **Figures 3**–**6**. More information regarding collection vouchers can be found in **Supplementary Table S1**.

To reconstruct the phylogenetic relationship of *Phtheirospermum*, 68 accessions from Orobanchaceae (including *Rehmannia*) were sampled, representing all five recognized tribes, 22 genera and 30 taxa (**Supplementary Table S1**). For *Ph. japonicum*, we sampled nine individuals from nine populations in Yunnan, Sichuan, Henan, Zhejiang, and Liaoning Provinces in China, and from Kanagawa Prefecture in Japan. We sampled 13 individuals of *Ph. tenuisectum*, three individuals of *Ph. muliense*, and four and eight individuals of two undescribed taxa, respectively. Geographic sampling of the *Ph. tenuisectum* complex is presented in **Figure 2**. We sampled the three recognized species of *Pterygiella* including seven individuals to test the relationship between the *Ph. tenuisectum* complex and *Pterygiella*. We included sequences for *Ps. glandulosa*, and the samples T8 (*J. S. Ying 4144*), L1 (*S. G. Wu 3582*), and H1 (*L. Lu LJ377*) from the study of Dong et al. (2013). Forty-nine samples were sequenced for this study. Thirty-six sequences of four DNA barcoding loci (nrITS, *matK*, *rbcL*, and *trnH-psbA*) from two *Pedicularis* samples (Yu et al., 2011) and seven *Pterygiella* samples (Dong et al., 2011) have been published, as well as two *trnL-F* sequences from *Pedicularis* (Yu et al., 2015a), and one from *Pterygiella* (Dong et al., 2011). Forty-six sequences extracted from seven published plastomes [*Bartsia inaequalis* Benth., *Castilleja paramensis* F. González & Pabón-Mora, *Cistanche phelypaea* (L.) Cout., *Lathraea squamaria* L., *Phelipanche purpurea* Soják, *Schwalbea americana* L., and *Striga hermonthica* (Delile) Benth.], and the available nrITS sequences, were included for phylogenetic analyses. Voucher information or GenBank accessions are presented in **Supplementary Table S1**.

### DNA Extraction, PCR Amplification, and Sequencing

Total genomic DNA was extracted from silica-gel-dried leaves using a modified CTAB protocol (Doyle and Doyle, 1987). We amplified and sequenced one nuclear (ribosomal internal transcribed spacer, nrITS) and seven plastid markers (*matK*, *rbcL*, *rps2*, *rps16*, *trnK-matK*, *trnH-psbA*, and *trnL-F*). There was no overlap between *matK* and the *trnK-matK* regions. Primer information is listed in **Supplementary Table S2**. Polymerase chain reaction (PCR) amplification conditions were: one cycle at 94°C for 3min; 35 cycles of 94°C for 45 s, 53–55°C for 60 s and 72°C for 60 s; followed by 72°C for 5 min (Yu et al., 2011). PCR products were purified using ExoSAP-IT (Affymetrix, Santa Clara, CA, United States). Sequencing reactions were performed using the ABI Prism BigDye Terminator Kits (Applied Biosystems, Inc.), following a modified protocol (Yu et al., 2011). Automated sequencing was performed on ABI 3730xl DNA sequencer (Applied Biosystems) in Kunming Institute of Botany, Chinese Academy of Sciences.

### Sequence Assembly and Alignment

The newly obtained raw sequences were assembled and edited using Geneious (Biomatters, Inc.) version 7.1 (Kearse et al., 2012). Assembled sequence consensuses were automatically aligned using MAFFT version 7.2 (Katoh and Toh, 2010), then adjusted manually in Geneious. The aligned matrices were concatenated using SequenceMatrix version 1.73 (Vaidya et al., 2011). Sequence characteristics were calculated using MEGA version 6.0 (Tamura et al., 2013).

### Phylogenetic Analyses

Maximum Likelihood (ML) and Bayesian Inference (BI) methods were used to reconstruct phylogenetic trees. The nrITS and the concatenated plastid datasets were analyzed separately. No nucleotide positions were excluded from analyses. The ML tree searches and bootstrap estimation of clade support were conducted with RAxML version 8.2.10 (Stamatakis et al., 2008). These analyses used the GTR substitution model with gamma-distributed rate heterogeneity among sites and the proportion of invariable sites estimated from the data. The concatenated plastid dataset was partitioned by gene. Support values for the node and clade were estimated from 1000 bootstrap replicates. Bootstrap supports (BS) ≥ 70 were considered well supported (Hillis and Bull, 1993). Partitioned BI analyses were performed using MrBayes version 3.2.6 (Ronquist and Huelsenbeck, 2003), with DNA substitution models selected for each gene partition by the Bayesian information criterion (BIC) using jModeltest version 2.1.10 (Guindon and Gascuel, 2003; Darriba et al., 2012). Markov Chain Monte Carlo (MCMC) analyses were run in MrBayes for 10,000,000 generations for each dataset, with two simultaneous runs, and each run comprising four incrementally heated chains. The BI analyses started with a random tree, and sampled every 1000 generations. The number of generations for the datasets was considered sufficient when the average standard deviation of split frequencies was lower than 0.005, and Potential Scale Reduction Factor (PSRF) of Convergence Diagnostic (Gelman and Rubin, 1992) was 1.00. The first 25% of the trees was discarded as burn-in, and the remaining trees were used to generate a majority-rule consensus tree. Clades recovering posterior probability values (PP) ≥ 0.95 were considered as well supported (Alfaro et al., 2003; Erixon et al., 2003; Kolaczkowski and Thornton, 2007). Both ML and BI analyses, as well as jModeltest, were performed at the CIPRES Science Gateway^1^.

Phylogenetic incongruence between ML and BI analyses of each dataset was visually compared using TreeGraph version 2.12 (Stover and Muller, 2010). The incongruence length difference test (ILD) (Farris et al., 1995) was not used to assess topological conflict between the nuclear and the concatenated plastid datasets because this analysis has been shown to be misleading (Baker et al., 2011). We used a conservative PP ≥ 0.95 and BS ≥ 70 as thresholds for identifying strongly incongruent clades between nrITS and the plastid datasets. In addition, topological incongruence between nrITS and the plastid datasets was also investigated using the Shimodaira–Hasegawa (SH) test (Shimodaira and Hasegawa, 1999) and the approximately unbiased (AU) test (Shimodaira, 2002). Topologies were constrained using Mesquite version 3.2 (Maddison and Maddison, 2017). The SH and AU tests were performed using PAUP version 4.10 (Swofford, 2003).

### Evolutionary Network

The nrITS and the plastid datasets of the *Ph. tenuisectum* complex were combined to recover a phylogenetic network using SplitsTree version 4.14.3 (Huson and Bryant, 2006). *Pterygiella* samples were included as outgroups. The Neighbor-net model was chosen using the Kimura 2-parameter (K2P) distance and Ordinary Least Square Method. Bootstrap values for the respective splits were estimated from 1000 bootstrap replicates.

### Estimation of Divergent Times

There is no reliable fossil in Orobanchaceae to calibrate the phylogeny; divergence times were indirectly estimated from calibrating fossils from other families of flowering plants (Bremer et al., 2004; Magallón et al., 2015; Wang et al., 2015; Wikström et al., 2015). Due to inconsistency in taxonomic sampling and marker choice in different studies, the mean crown ages of Orobanchaceae varied from 26.0 Mya (Wikström et al., 2015) to 74.54 Mya (Wang et al., 2015). To improve the molecular dating on Orobanchaceae and the *Ph. tenuisectum* complex, we selected 24 families and 102 genera from Lamiales, and *Solanum tuberosum* as the outgroup (**Supplementary Table S3**). Two chloroplast DNA regions, *rps2*, and *trnK* were concatenated for molecular dating. Dating analyses were conducted using Markov Chain Monte Carlo (MCMC) methods in BEAST version 2.4 (Bouckaert et al., 2014), which was performed at the CIPRES Science Gateway^2^. For setting parameters of BEAUti, we chose “BEAST model test” for “Site model,” “Relaxed Clock Log Normal” for “Clock model,” and “Yule Model” for speciation. Meanwhile, we selected four fossils to calibrate stem nodes of four families using CladeAge package (Matschiner et al., 2017). Of them, Acanthaceae (*Acanthus rugatus* Reid & Chandler), Bignoniaceae (*Radermachera pulchra* Reid & Chandler), Lamiaceae (*Melissa parva* Reid & Chandler) have reliable fossils from the Bembridge Flora (England) (Reid and Chandler, 1926) of the Late Eocene (Collinson et al., 2010), and Oleaceae (*Fraxinus wilcoxiana* Berry) from the Claiborne Formation of western Tennessee (United States) of the Middle Eocene (Call and Dilcher, 1992). The stem age of Acanthaceae (C4), Bignoniaceae (C3), and Lamiaceae (C5) was set as 33.9–37.8 Mya, and that of Oleaceae (C2) as 41.2–47.8 Mya. In addition, we used a normal model to constrain the crown age for Lamiales (C1) and Orobanchaceae (C6) as 84 ± 10 Mya and 56 ± 10 Mya, respectively. These ages were adopted from the TimeTree website^3^ (Hedges et al., 2006). Three independent MCMC runs with the same parameters were conducted. Each MCMC ran 100,000,000 generations, and was sampled every 10,000 generations. The first 5,000 generations were removed as “Pre Burnin.” Three Log outputs of the BEAST analyses were jointly evaluated using Tracer version 1.6. Effective sample sizes (ESS) of all parameters were more than 200, indicating that MCMC sampling was adequate (Lanfear et al., 2016). Trees of three runs were combined using LogCombiner by burning 20% of the initial trees. The Maximum Clade Credibility (MCC) tree was generated using TreeAnnotator by setting “Mean heights” for the “Node heights”. The MCC tree was visualized using FigTree version 1.4.2^4^.

## Results

### Data Sets

To evaluate sequence matrix characteristics, we classified the samples of *Phtheirospermum* spp. and *Pterygiella* spp. in several groups. First, four major groups were adopted, i.e., *Phtheirospermum*, *Pterygiella*, the *Ph. tenuisectum* complex and the *Ph. tenuisectum* complex + *Pterygiella*. Then, every species with two or more samples in *Phtheirospermum* was treated as an independent group. Five species groups in *Phtheirospermum* were recognized, i.e., four groups in the *Ph. tenuisectum* complex, and *Ph. japonicum*.

Sequence characteristics of nrITS, seven plastid DNA loci, and the concatenated plastid datasets are presented in **Table 1**. The nrITS dataset was the most informative marker (except in the *Ph. muliense* and *Phtheirospermum* sp. 1), followed by two chloroplast intergenic spacers (*trnH-psbA* and *trnL-F*) and two introns (*matK-trnK*, and *rps16*). Of the three coding genes, *matK* was the most informative. Sequences of *Ph. japonicum* had no informative variation across seven plastid markers throughout nine populations. Only four deletions/insertions were found in the Japanese sample (J9), i.e., indels of a single nucleotide for *rps16* and *trnK*, and two nucleotides in *trnL-F*. In contrast, the sequences of *Ph. tenuisectum* showed the highest variation at the species level across all eight DNA regions. The remaining three taxa of the *Ph. tenuisectum* complex were restricted to the Hengduan Mountains region, and showed fewer variations. Though 4.40% of nrITS sites of *Pterygiella* were variable, only 0 to 0.29% of the sites across seven plastid loci were variable.

**Table 1:** Summary information for seven DNA markers.

	ITS	*matK*	*rbcL*	*rps2*	*rps16*	*trnH-psbA*	*trnK-matK*	*trnL-F*	cpDNA
Number of accessions	58	57	55	57	58	59	55	59	66
Aligned length (bp)	863	791	609	659	1024	982	1284	1024	6373
**Variable sites/Parsimony informative sites**									
All samples	394/295	311/141	114/58	250/120	379/186	451/273	436/211	422/198	2365/1199
*Phtheirospermum*	149/140	40/39	14/13	11/11	55/53	59/58	61/58	52/47	292/279
*Ph. japonicum*	4/1	0/0	0/0	0/0	0/0	0/0	0/0	0/0	0/0
*Ph. tenuisectum* complex + *Pterygiella*	93/73	15/10	5/4	4/3	22/18	36/35	19/13	23/16	124/99
*Pterygiella*	38/31	3/0	0/0	1/0	3/0	2/0	3/0	2/0	14/0
*Ph. tenuisectum* complex	62/48	11/9	5/4	3/3	14/11	32/32	15/12	19/14	99/85
*Ph. tenuisectum*	19/6	5/3	2/2	0/0	5/3	14/14	5/4	6/2	37/28
*Ph. muliense*	0/0	0/0	0/0	0/0	1/0	0/0	0/0	1/0	1/0
*Phtheirospermum* sp. 1	1/0	1/1	2/0	0/0	0/0	0/0	0/0	1/0	4/1
*Phtheirospermum* sp. 2	7/2	0/0	1/0	0/0	1/0	4/0	4/2	3/2	13/4

### Phylogenetic Analyses Using nrITS and the Plastid Datasets

**FIGURE 3:**
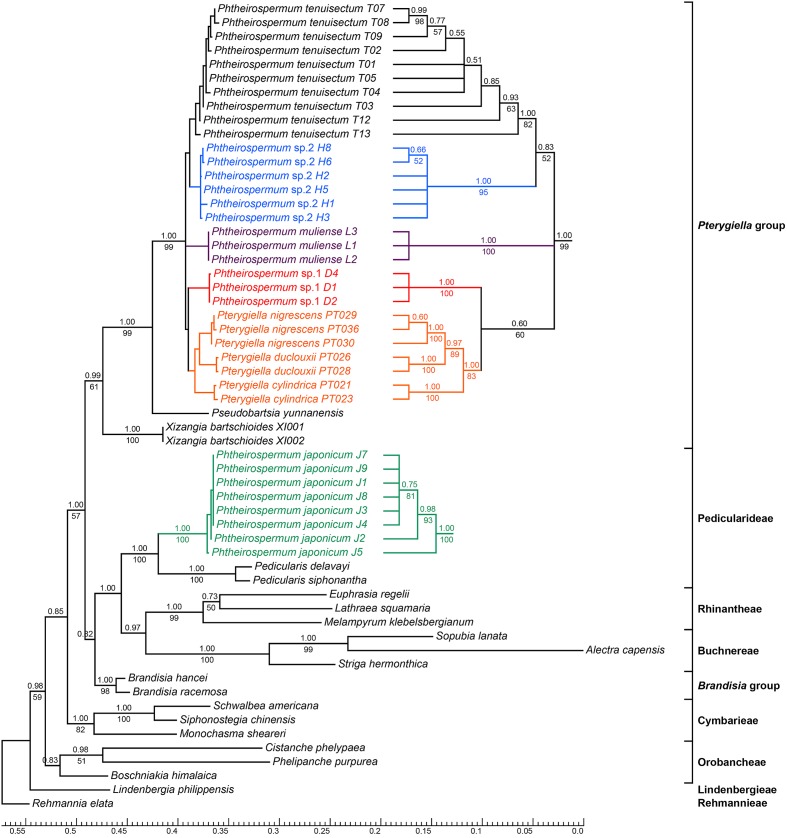
Bayesian inference (BI) tree inferred from the nrITS dataset. ML Bootstrap values are presented under branches, and BI posterior probabilities are shown above branches. The topology of some clades with short branch lengths appear on the right. The bottom scale bar represents the number of substitutions per site.

**FIGURE 4:**
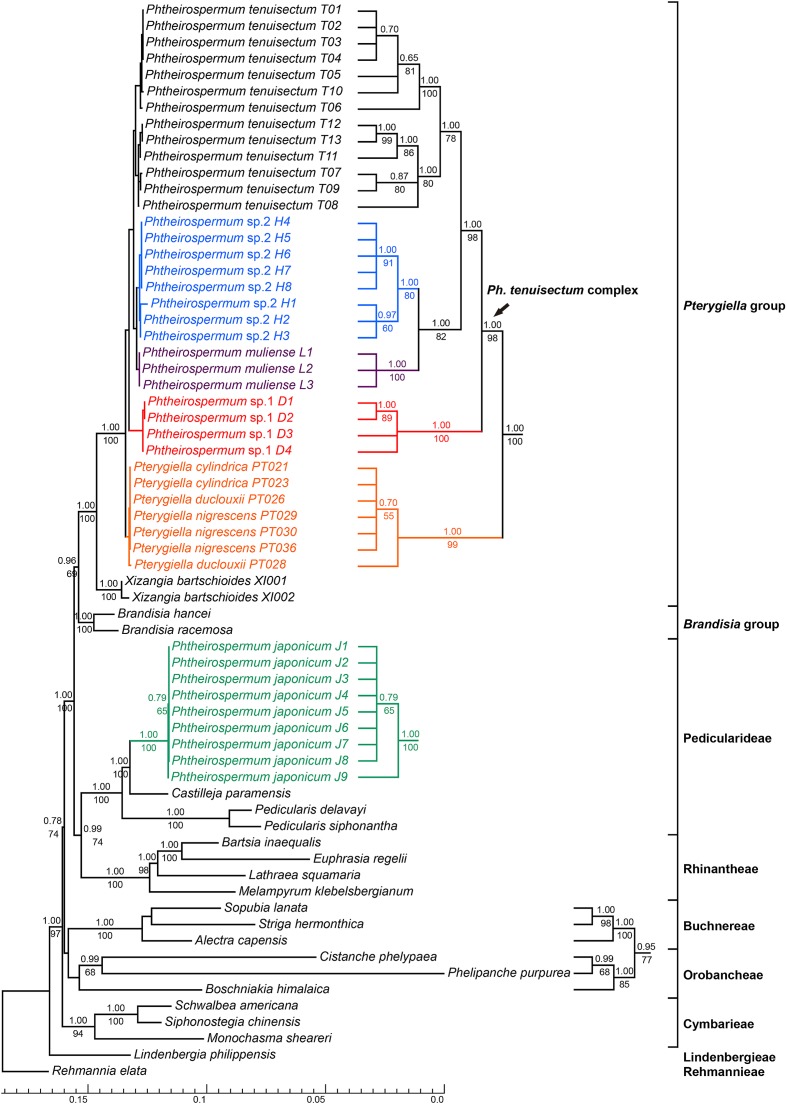
Bayesian inference tree inferred from the combined plastid dataset. The seven plastid markers include *matK*, *rbcL*, *rps2*, *rps16*, *trnK-matK*, *trnH-psbA* and *trnL-F*. ML Bootstrap values are presented under branches, and BI posterior probabilities are shown above branches. The topology of some clades with short branch lengths appear on the right. The bottom scale bar represents the number of substitutions per site.

Phylogenetic trees using nrITS and the plastid datasets are presented in **Figures 3**, **4**, respectively. *Pterygiella* + *Phtheirospermum* + *Xizangia* (hereafter referred to as the *Pterygiella* group) was well supported as monophyletic (nrITS: BS/PP = 61/0.99; plastid: BS/PP = 100/1.00), and not nested in any other formerly recognized tribe. The plastid dataset supported *Brandisia* spp. as sister to this group (BS/PP = 69/0.96). None of the analyses resolved *Pterygiella* group close to tribe Rhinantheae.

Both nrITS and the plastid datasets recovered *Ph. japonicum* nested within Pedicularideae (nrITS and plastid: BS/PP = 100/1.00). The *Ph. tenuisectum* complex and *Pterygiella* spp. also formed a well-supported clade in both analyses (nrITS: BS/PP = 99/1.00; plastid: BS/PP = 100/1.00). The four morphotypes of the *Ph. tenuisectum* complex were recovered as reciprocally monophyletic, well-supported lineages. The *Ph. tenuisectum* complex was strongly supported as monophyletic by the plastid dataset (BS/PP = 99/1.00), and phylogenetic relationships for the four morphotypes were fully resolved (**Figure 4**). In contrast, *Pterygiella* spp. were nested within the *Ph. tenuisectum* complex in nrITS analyses (BS/PP = 100/1.00). In this analysis, *Ph. tenuisectum* was supported as sister to *Phtheirospermum* sp. 2 (BS/PP = 59/0.92), and *Phtheirospermum* sp. 1 and *Pterygiella* spp. formed a clade (BS/PP = 60/0.60).

Based on support values, we found hard topological conflicts between nrITS and the plastid datasets in the placement of tribes Orobancheae and Buchnereae. Additionally, these placements disagree with those recovered by McNeal et al. (2013). Further topological comparisons using SH and UA tests are presented in **Table 2**. In constrained analyses using nrITS dataset, both SH and UA tests indicated that the monophyly of the *Ph. tenuisectum* complex and *Brandisia* spp. sister to *Pterygiella* group were supported (*P* > 0.2). On the other hand, sister relationship between tribes Orobancheae and Buchnereae, and tribes Rhinantheae and Pedicularideae were rejected (*P* < 0.05). The constrained analyses using the plastid data rejected the paraphyly of the *Ph. tenuisectum* complex by the AU test (*P* < 0.05) and the sister relationship between tribes Buchnereae and Rhinantheae by both SH and UA tests. Therefore, nrITS and the plastid datasets should be analyzed separately.

**Table 2:** Summary of the Shimodaira-Hasegawa (SH) and the approximately unbiased (AU) tests.

	Ln likelihood	∂	SH	AU
**nrITS analyses compared with constraint clades from plastid genes analyses**				
Unconstrained nrITS analysis	8055.53966			
(A,(B,(C,((G,(E,(D,F))),(H,(I,(J2,(J3,(J1,J4)))))))))	8059.23895	3.7093	0.6969	0.2106
(A,(B,(C,((G,(E,(D,F))),(H,(I,(J3,(J1,(J2,J4)))))))))	8062.73186	7.2022	0.4530	0.2702
(A,(B,(C,((E,(D,F)),(G,(H,(I, (J2,(J3,(J1,J4))))))))))	8062.60097	7.0713	0.5329	0.2203
(A,(B,((C,D),((E,F),(G,(H,(I, (J2,(J3,(J1,J4))))))))))	8086.13276	30.6031	**0.0430**	**0.0218**
**Plastid-gene analyses compared with constraint clades from nrITS analyses**				
Unconstrained five-plastid-gene analysis	32428.95009			
(A,(B,((C,D),((E,F),(G,(H,((I,J3),(J1,(J2,J4)))))))))	32445.35123	16.4011	0.3100	**0.0100**
(A,(B,((C,D),((E,F),(G,(H,((I,J1,J3,(J2,J4)))))))))	32443.70799	14.7579	0.3457	**0.0437**
(A,(B,(C,((E,(D,F)),(G,(H,((I,J1,J3,(J2,J4))))))))))	32505.62965	76.6796	**0.0003**	**0.0000**
(A,(B,(C,((G,(E,(D,F))),(H,((I,J1,J3,(J2,J4)))))))))	32513.13974	84.1897	**0.0003**	**0.0000**

*P-values less than 0.05 appear in boldface. Log likelihood scores for the unconstrained analysis are given, as well as the difference in log likelihood scores between the unconstrained and the constraint topologies (∂). A, Rehmannia + Lindenbergia; B, Cymbarieae; C, Orobancheae; D, Buchnereae; E, Pedicularideae; F, Rhinantheae; G, Brandisia spp.; H, Xizangia + Pseudobartsia; I, Pterygiella spp.; J, Phtheirospermum tenuisectum complex; J1, Ph. tenuisectum; J2, Ph. muliense; J3, Phtheirospermum sp. 1; J4, Phtheirospermum sp. 2.*

### Phylogenetic Network of the *Phtheirospermum tenuisectum* Complex

**FIGURE 5:**
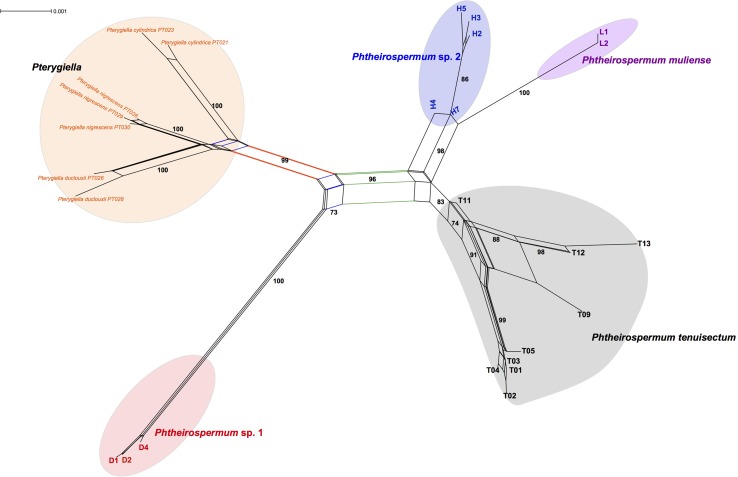
Phylogenetic network of the *Ph. tenuisectum* complex and *Pterygiella* spp. using all loci. The scale bar on the top left represents the number of substitutions per site.

To further explore phylogenetic incongruence within the *Ph. tenuisectum* complex, a phylogenetic network was inferred from the total dataset (**Figure 5**). A well supported split separated the *Ph. tenuisectum* complex from *Pterygiella* spp. (BS = 99). Each of the three species of *Pterygiella* were well resolved as monophyletic (BS = 100). The four morphotypes of the *Ph. tenuisectum* complex were also recovered as separate clusters. This pattern of relationships is similar to the plastid phylogeny, in which that *Ph. tenuisectum* was sister to *Phtheirospermum* sp. 2 + *Ph. muliense* (BS = 96). In addition, *Phtheirospermum* sp. 1 had connections with *Pt. duclouxii* + *Pt. nigrescens* (BS = 73), likely based on signal from the nrITS dataset.

### Estimation of Divergence Times

**FIGURE 6:**
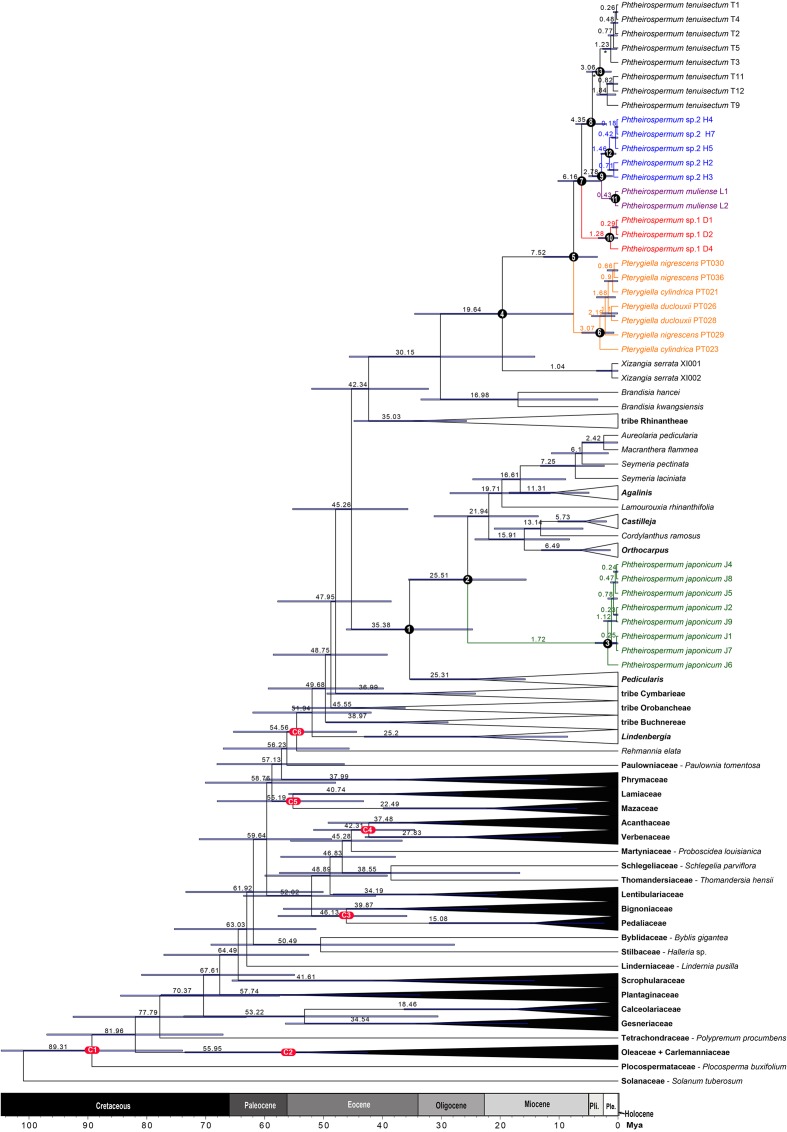
A simplified maximum clade credibility tree of Lamiales from BEAST divergence time analysis. The estimated age of nodes is presented above the branch. Node bars represent the 95% highest posterior density (HPD) interval. Six calibrated (red) and 13 key stem/crown nodes (black) were annotated by letters and/or numbers.

The MCC tree is shown in **Figure 6** and **Supplementary Figure S1**, including mean ages and 95% HPD interval bars. The exact values for six calibrated and 13 annotated nodes are summarized in **Table 3**. The mean substitution rate was 1.10 × 10^-3^ per site per million years (95% HPD: 9.04 × 10^-4^ - 1.29 × 10^-3^). The Yule speciation rate was 4.65 × 10^-2^ (95% HPD: 3.63 × 10^-2^ - 5.67 × 10^-2^). The coefficient of variation was 1.08 (95% HPD: 0.91 - 1.27), indicating that a high degree of rate heterogeneity was observed across the tree and that a relaxed-clock model was suitable for this dataset (Drummond et al., 2006).

**Table 3:** Estimated age, 95% highest posterior density (HPD) interval, and posterior probability (PP) value of six calibrated and 13 key stem/crown nodes.

Nodes	Calibration ages (Mya)	Estimation		
		Mean (Mya)	95% HPD (Mya)	PP
C1: Lamiales crown	84 ± 10	89.31	76.06 - 104.75	1.00
C2: Oleaceae stem	41.2 - 47.8	55.95	42.45 - 73.55	1.00
C3: Bignoniaceae stem	33.9 - 37.8	46.13	35.84 - 57.73	0.65
C4: Acanthaceae stem	33.9 - 37.8	42.31	34.51 - 51.69	0.81
C5: Lamiaceae stem	33.9 - 37.8	55.19	43.19 - 68.06	0.86
C6: Orobanchaceae crown	56 ± 10	54.56	44.37 - 65.30	1.00
1: Pedicularideae crown		35.38	24.69 - 46.14	1.00
2: *Ph. japonicum* stem		25.51	15.58 - 35.56	1.00
3: *Ph. japonicum* crown		1.72	0.29 - 3.88	1.00
4: *Pterygiella* group crown		19.64	7.62 - 34.54	0.99
5: *Ph. tenuisectum* lineage stem		7.52	3.50 - 12.64	1.00
6: *Pterygiella* crown		3.07	0.69 - 6.16	0.99
7: *Ph. tenuisectum* complex crown		6.16	2.91 - 10.21	0.95
8: *Ph. tenuisectum* + *Ph.* sp. 2 + *Ph. muliense* crown		4.35	1.96 - 7.19	1.00
9: *Ph. Muliense* + *Ph.* sp. 2 crown		2.78	0.95 - 4.97	1.00
10: *Phtheirospermum* sp. 1 crown		1.28	0.05 - 3.35	1.00
11: *Ph. muliense* crown		0.43	0.00 - 1.44	1.00
12: *Phtheirospermum* sp. 2 crown		1.46	0.33 - 2.91	1.00
13: *Ph. tenuisectum* crown		3.06	1.14 - 5.34	1.00

*The calibration ages of the six nodes are also listed. The calibration mean ages of Lamiales (C1) and Orobanchaceae (C6) crown were from TimeTree website (Hedges et al., 2006), and those of Acanthaceae, Bignoniaceae, Lamiaceae, and Oleaceae stem were based on macrofossils (Reid and Chandler, 1926; Call and Dilcher, 1992; Collinson et al., 2010).*

Familial relationships in Lamiales were not fully resolved, and only some nodes were well supported (PP ≥ 0.95). Paulowniaceae and Phrymaceae were strongly supported as sister to Orobanchaceae (PP = 1.00). In Orobanchaceae, nine clades were well resolved (PP ≥ 0.99). Of these, seven clades have been recognized as tribes, and *Brandisia* as an independent lineage (Stevens, 2001 onward; McNeal et al., 2013). The *Pterygiella* group was recovered as monophyletic. Tribe Rehmannieae was the most basal clade, followed by splits marking the divergences of tribes Lindenbergieae and Buchnereae from the remainder of the lineage. The following splits mark the divergences of tribes Orobancheae and Cymbarieae successively, followed by Pedicularideae and Rhinantheae, leaving *Brandisia* and the *Pterygiella* group as a strongly supported clade (PP = 1.00). *Brandisia* was weakly supported as sister to the *Pterygiella* group. Within the *Pterygiella* group, the *Ph. tenuisectum* complex was strongly supported as monophyletic, and phylogenetic relationships of four morphotypes were well resolved.

The divergence time estimate for Orobanchaceae was in the early Eocene (54.56 Mya). Tribe Pedicularideae separated from tribe Rhinantheae – the *Pterygiella* group at the mid-Eocene (45.26 Mya), then *Ph. japonicum* diverged from the new world genera of tribe Pedicularideae at the late Oligocene (25.51 Mya), and its subsequent diversification occurred at the mid-Pleistocene (1.72 Mya). Diversification of the *Pterygiella* group was estimated to be around the early Miocene (19.64 Mya). The *Ph. tenuisectum* complex diverged from *Pterygiella* spp. during the later Miocene (7.52 Mya), with the four morphotype lineages fully established by the early Pleistocene (2.78 Mya).

## Discussion

### Phylogeny of Orobanchaceae

The current delimitation of Orobanchaceae includes *Rehmannia* and *Trianeophora* as the basal tribe, i.e., Rehmannieae (Stevens, 2001 onward), which was strongly supported by phylogenetic analyses (Xia et al., 2009; Zeng et al., 2017). To date, seven tribes and *Brandisia* have been recognized in Orobanchaceae (Stevens, 2001 onward; McNeal et al., 2013). Although *PHYA* and *PHYB* nuclear data strongly supported *Pterygiella* as sister to the remaining genera of tribe Rhinantheae; both nrITS and the combined *rps2* and *matK* datasets poorly supported *Pterygiella* as sister to *Brandisia*, and only the plastid dataset weakly supported the clade *Pterygiella* + *Brandisia* as sister to the remaining genera of tribe Rhinantheae (McNeal et al., 2013). In this study, both nrITS and the plastid datasets strongly resolved the *Pterygiella* group as monophyletic. The plastid dataset strongly supported *Brandisia* spp. as sister to the *Pterygiella* group; however, nrITS weakly resolved *Brandisia* as sister to a clade comprising tribes Buchnereae, Pedicularideae, and Rhinantheae. Therefore, *Brandisia* and the *Pterygiella* group should be treated as two separate groups or tribes in Orobanchaceae.

### Polyphyly of *Phtheirospermum*

All phylogenetic analyses recovered polyphyly of *Phtheirospermum*, e.g., *Ph. japonicum* grouped with tribe Pedicularideae and the *Ph. tenuisectum* complex was close to *Pterygiella* spp. in the clade including *Pseudobartsia* and *Xizangia* (**Figures 3**, **4**). This is not surprising, given that previous analyses place *Ph. japonicum* within Pedicularideae (Bennett and Mathews, 2006; McNeal et al., 2013; Yu et al., 2015a; Pinto-Carrasco et al., 2017). In this tribe, *Pedicularis* is the most basal group, followed by *Ph. japonicum*. The remaining genera are mainly distributed in the New World (Bennett and Mathews, 2006; McNeal et al., 2013), including *Castilleja*, *Orthocarpus*, and *Triphysaria*, another important model for parasitic plant genetics, genomics, and evolution (Tomilov et al., 2005; Westwood et al., 2010; Bandaranayake et al., 2012; Yang et al., 2015). The *Ph. tenuisectum* complex is clearly excluded from tribe Pedicularideae, forming a lineage including *Pterygiella*, *Xizangia*, and *Pseudobartsia*. The taxonomic confusion of *Phtheirospermum* can be ascribed to J. D. Hooker who described the second species *Ph. parishii* in *Phtheirospermum*, and transferred *Euphrasia glandulosa* Benth. to this genus (Hooker and Thomson, 1884). Based on the ellipsoid seeds and the minutely reticulated seed surface, C. B. Clarke suggested treating *Ph. parishii* as a separate genus (i.e., “*Emmenospermum*”), but Hooker did not adopt this treatment, because seed morphology of Scrophulariaceae was high variable within and among genera (Hooker and Thomson, 1884). Based on Hooker’s delimitation of *Phtheirospermum*, *Ph. tenuisectum* and *Ph. muliense* were placed in this genus (Bureau and Franchet, 1891; Tao, 1996). However, the phylogenetic analyses demonstrate that the *Ph. tenuisectum* complex should be separated from *Ph. japonicum*. Furthermore, this separation is justified by morphology; compared to *Ph. japonicum*, members of the *Ph. tenuisectum* complex bear linear rather than ovate to orbicular pinnae, smaller flowers with yellow rather than red corollas, and smaller fruits and seeds.

Though the *Ph. tenuisectum* complex was strongly supported as monophyletic by the plastid dataset (**Figure 4**), nrITS resolved *Ph. tenuisectum* complex as paraphyletic, with *Pterygiella* nested within it (**Figure 3**). Pinto-Carrasco et al. (2017) have combined the *Ph. tenuisectum* complex with *Pterygiella* in a single taxon, creating new combinations *Pt. muliensis* (C.Y.Wu & D.D.Tao) Pinto-Carrasco, E.Rico & M.M.Mart.Ort., *Pt. parishii* (Hook. f.) Pinto-Carrasco, E.Rico & M.M.Mart.Ort., and *Pt. tenuisecta* (Bureau and Franch.) Pinto-Carrasco, E.Rico & M.M.Mart.Ort.. This taxonomic decision was justified on the basis of phylogenetic results, and morphological evidence including five-toothed calyces and pollen characters. However, we suggest that the *Ph. tenuisectum* complex and *Pterygiella* should be retained as separated groups. First, in the constrained analyses, monophyly of the *Ph. tenuisectum* complex was not rejected (**Table 2**). Moreover, the *Ph. tenuisectum* complex is morphologically quite distinct from *Pterygiella*. *Pterygiella* spp. are characterized by winged stems (except *Pt. cylindrica* P. C. Tsoong), lanceolate and entire leaves, and by a calyx that is broadly campanulate and 5-veined (**Supplementary Figure S2**). Conversely, the *Ph. tenuisectum* complex has cylindrical stems, pinnatisect leaves, and a tubular calyx. In addition, seed morphology and capsule indumentum also differ between *Pterygiella* and the *Ph. tenuisectum* complex (Dong et al., 2013, 2015). Therefore, we suggest treating the *Ph. tenuisectum* complex as an independent genus. So far, there is no available name for this lineage. The name “*Emmenospermum*” cannot be selected because it would be a later homonym of *Emmenosperma* F. Mueller (Rhamnaceae). A new genus name needs to be published in accordance with the International Code of Nomenclature for algae, fungi, and plants (McNeill et al., 2012), including comprehensive morphological comparison with *Pterygiella* spp. and other relatives.

### Taxonomic Significance and Evolution of *Phtheirospermum japonicum*

The genus *Phtheirospermum* was established on the basis of *Ph. chinense* Bunge, which was collected in north China (Fischer and Meyer, 1835). Both the genus and species names are ascribed to A.A. Bunge in F.E.L. Fischer’s and C.A. Meyer’s edited monograph, Index Seminum [St. Petersburg]. However, an early name *Gerardia japonica* Thunberg had been described based on a Japanese specimen. Therefore, *Ph. japonicum* is the correct name to replace *Ph. chinense* (Kanitz and Weiss, 1878). Our phylogenetic results showed the polyphyly of *Phtheirospermum*, the current delimitation of *Phtheirospermum* include *Ph. japonicum* alone, and *Ph. tenuisectum* complex needs to be transferred to a new genus. Currently, *Ph. japonicum* has been established as the experimental model for the study of genetics and development of the haustorium (Cui et al., 2016; Ishida et al., 2016). Maintaining the name *Ph. japonicum* will therefore benefit molecular biologists using this model in publications or communications with public audiences and plant specialists. This taxonomic decision would forestall confusion as has arisen in the nomenclature of the monkeyflower (*Mimulus guttatus* DC.), a model organism for studies of evolution and ecology that was shown to be polyphyletic. The correct name of the monkeyflower was changed to *Erythranthe guttata* (DC.) G. L. Nesom (Barker et al., 2012). However, some recent publications still use the old name *M. guttatus* (Lee et al., 2016; Ferris et al., 2017; Puzey et al., 2017).

*Phtheirospermum japonicum* is a widely distributed species in Eastern Asia (Hong et al., 1998). In this study, we sampled nine individuals from southwestern (Yunnan: J2, J3, J7, and J8; Sichuan: J6), through central China (Henan: J5), to eastern (Zhejiang: J1) and northeastern China (Liaoning: J4), and extending to Japan (Kanagawa: J9). Both nrITS and the plastid evidence indicated that genetic variation in *Ph. japonicum* was low, and current distribution range might be a result of a recent population expansion. The results of molecular dating showed that a recent radiation occurred at around 1.72 Mya (95% HPD: 0.29–3.88 Mya), and the Japanese sample was derived from a Yunnan sample (J2) at around 0.23 Mya (95% HPD: 0.0–0.73 Mya).

### Cryptic Speciation of *Phtheirospermum tenuisectum* Complex in the Hengduan Mountains Region

The *Ph. tenuisectum* complex can be classified as four species lineages, each strongly supported by both nrITS and the plastid datasets (**Figures 3**, **4**), and diagnosable by morphological characters (**Figure 1**). Phylogenetic relationships among the four lineages were inconsistent between nrITS and the plastid datasets. It is likely that because resolution of the nrITS tree was low, constrained analyses using nrITS dataset did not reject the constrained plastid topology (**Table 2**). The plastid dataset strongly supported that *Phtheirospermum* sp. 1 was the sister to the three other lineages, in which *Phtheirospermum* sp. 2 was sister to *Ph. muliense*. As an ancestral lineage in the *Ph. tenuisectum* complex, *Phtheirospermum* sp. 1 might share plesiomorphic characters with *Pterygiella* spp. (i.e., incomplete lineage sorting), so that the nrITS dataset weakly supported *Pterygiella* spp. as sister to *Phtheirospermum* sp. 1 (**Figure 3**), and phylogenetic network showed a well-supported genetic connection between *Phtheirospermum* sp. 1 and *Pt. duclouxii* + *Pt. nigrescens* (**Figure 5**). Meanwhile, ancient hybridization/introgression cannot be excluded between *Pterygiella* spp. and *Phtheirospermum* sp. 1. In addition, ancient hybridization/introgression and incomplete lineage sorting may also have occurred between *Phtheirospermum* sp. 2 and *Ph. tenuisectum*.

The last two uplifts of the Qinghai–Tibet Plateau happened at around 8.0–7.0 Mya and 3.6–1.5 Mya (Zheng et al., 2000; Garzione et al., 2003; Molnar, 2005; Ge et al., 2006; Li et al., 2015; but see Renner, 2016). Many species groups diversified rapidly with these uplifts in the Hengduan Mountains (Wen et al., 2014; Xing and Ree, 2017). In this study, the estimated ages for the origin of *Ph. tenuisectum* complex and its subsequent divergences fell into the range of the two uplifts (**Table 3** and **Figure 6**). The uplift during 8.0–7.0 Mya may have driven divergence of the *Ph. tenuisectum* complex from *Pterygiella*. The center of diversity for both the *Ph. tenuisectum* complex and *Pterygiella* is in northwestern Yunnan and southwestern Sichuan, perhaps the place of origin of the *Ph. tenuisectum* complex. Overlapping species distributions among these genera out of the Hengduan Mountains may be the results of later population expansion. Of the four lineages in the *Ph. tenuisectum* complex, *Ph. muliense*, *Phtheirospermum* sp. 1, and *Phtheirospermum* sp. 2 grow along/near the dry valleys of Jinsha River and its tributaries, with only the sample H5 of *Phtheirospermum* sp. 2 extend to Lancang River (**Figure 2**). In contrast, *Ph. tenuisectum* mainly occurs in meadows and has a wider distribution. The valleys of Jinshan and Lancang Rivers and their tributaries in the Hengduan Mountains region experience a specialized dry-hot valley climate (Yang et al., 2016), which may have contributed to divergence of *Ph. muliense*, *Phtheirospermum* sp. 1, and *Phtheirospermum* sp. 2. To address the evolutionary history of *Ph. tenuisectum* complex in the future, a phylogeographic approach may be applied using more dense population samplings and multiple individuals per population.

## Author Contributions

W-BY, HW, and D-ZL conceived the study. W-BY, CR, LL, HW, and RC collected the data. W-BY analyzed the data. W-BY, CR, LL, CdP, and D-ZL interpreted the results. All authors wrote and revised the paper, and approved the final version.

## Conflict of Interest Statement

The authors declare that the research was conducted in the absence of any commercial or financial relationships that could be construed as a potential conflict of interest.

## Footnotes

1 http://www.phylo.org

2 http://www.phylo.org

3 http://timetree.org/

4 http://tree.bio.ed.ac.uk/software/figtree/

## Supplementary Material

FIGURE S1 A detailed maximum clade credibility tree of Lamiales from BEAST divergence time analysis. The estimated age of nodes was presented above the branch. Node bars represents 95% highest posterior density (HPD) interval. PP ≥ 95% is indicated by one asterisk (^∗^), and 95% ≥ PP ≥ 75% is indicated by two asterisks under the branch. Six calibrated (red) and 13 key stem/crown nodes (black) were annotated by letters and/or numbers.

FIGURE S2 Photographs of *Pterygiella*. **(A–C)** *Pterygiella cylindrica* P. C. Tsoong; **(D–F)** *Pt. duclouxii* Franchet; and **(G–I)** *Pt. nigrescens* Oliver.

TABLE S1 Voucher information for phylogeny of *Phtheirospermum* and *Pterygiella*, including 20 other genera of Orobanchaceae.

TABLE S2 Primer information for PCR amplification and sequencing in this study.

TABLE S3 Voucher information for molecular dating of Orobanchaceae, including selected genera and families from Lamiales.
