# Supplementary figures and images for: The Hemiparasitic Plant Phtheirospermum (Orobanchaceae) Is Polyphyletic and Contains Cryptic Species in the Hengduan Mountains of Southwest China
Source: Front Plant Sci. 2018 Feb 9;9:142. doi: 10.3389/fpls.2018.00142 (PMC5812252; doi:10.3389/fpls.2018.00142)

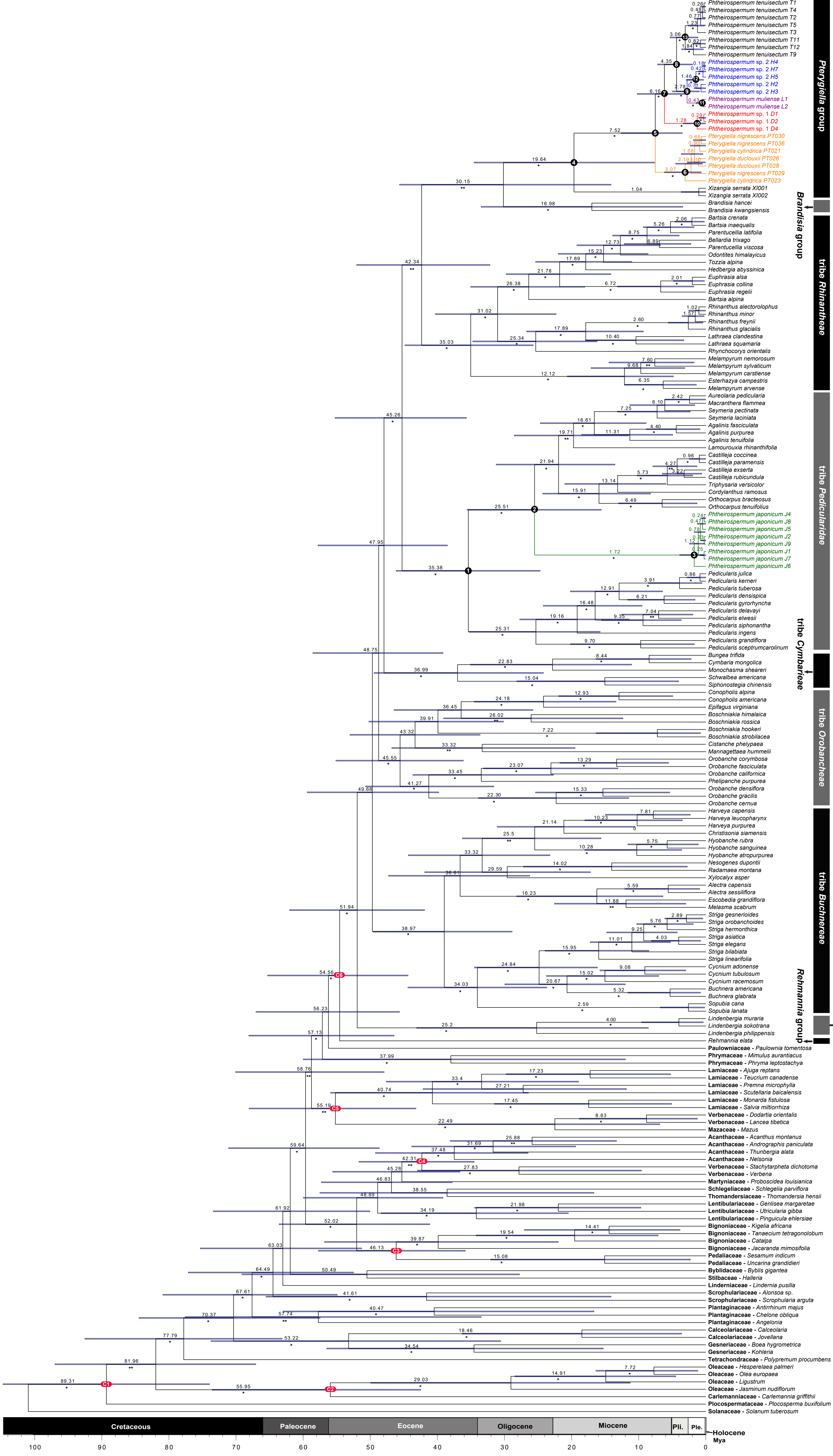

Supplement: FIGURE S1 — A detailed maximum clade credibility tree of Lamiales from BEAST divergence time analysis. The estimated age of nodes was presented above the branch. Node bars represents 95% highest posterior density (HPD) interval. PP ≥ 95% is indicated by one asterisk (∗), and 95% ≥ PP ≥ 75% is indicated by two asterisks under the branch. Six calibrated (red) and 13 key stem/crown nodes (black) were annotated by letters and/or numbers. [file Image_1.PDF]

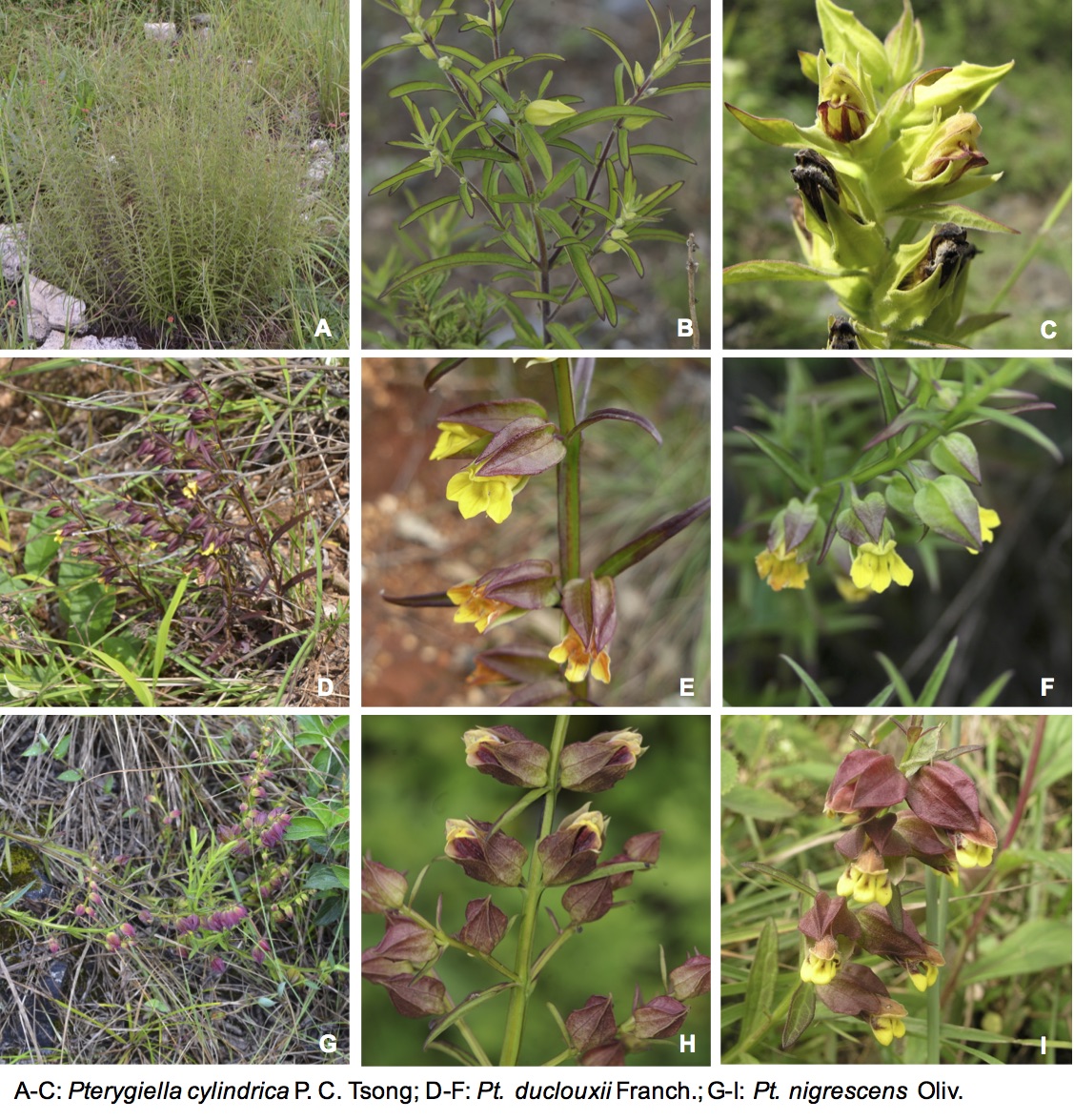

Supplement: FIGURE S2 — Photographs of Pterygiella. (A–C) Pterygiella cylindrica P. C. Tsoong; (D–F) Pt. duclouxii Franchet; and (G–I) Pt. nigrescens Oliver. [file Image_2.JPEG]
