# Supplementary material for: The Hemiparasitic Plant Phtheirospermum (Orobanchaceae) Is Polyphyletic and Contains Cryptic Species in the Hengduan Mountains of Southwest China
Source: Front Plant Sci. 2018 Feb 9;9:142. doi: 10.3389/fpls.2018.00142 (PMC5812252; doi:10.3389/fpls.2018.00142)
Supplement: TABLE S2 — Primer information for PCR amplification and sequencing in this study. [file Table_2.DOCX]

**Table S2.** Primer information for PCR amplification and sequencing in this study.

| Primer name | Sequences | References |
| --- | --- | --- |
| ITS | 18sF: ACCGATTGAATGGTCCGGTGAAGTGTTCG 26sR: CTGAGGACGCTTCTCCAGACTACAATTCG | (Gruenstaeudl et al. 2009) |
| *matK* | 3F_KIM: CGTACAGTACTTTTGTGTTTACGAG 1R_KIM: AATATCCAAATACCAAATCC | (Hollingsworth et al. 2009) |
| *rbcL* | 1F: ATGTCACCACAAACAGAAAC 724R: TCGCATGTACCTGCAGTAGC | (Fay et al. 1997) |
| *rps16* | Rpsf: GTGGTAGAAAGCAACGTGCGACTT RpsR2: TCGGGATCGAACATCAATTGCAAC | (Oxelman et al. 1997) |
| *rps2* | rps2-661R: ACCCTCACAAATAGCGAATACCAA rps2-18F: GGRKARAAATGACAAGAAGATATTGG | (dePamphilis et al. 1997) |
| *trmK-matK* | trnK11: CTCAACGGTAGAGTACTCG matK510r: GAAGAGTTTGAACCAAKAYTT | (Young et al. 1999) |
| *trnH-psbA* | trnH2: CGCGCATGGTGGATTCACAATCC psbAF: GTTATGCATGAACGTAATGCTC | (Sang et al. 1997; Tate and Simpson 2003) |
| *trnL-F* | trnc: CGAAATCGGTAGACGCTACG trnf: ATI'TGAACTGGTGACACGAG | (Taberlet et al. 1991) |

**References**

dePamphilis CW, Young ND, Wolfe AD (1997) Evolution of plastid gene rps2 in a lineage of hemiparasitic and holoparasitic plants: Many losses of photosynthesis and complex patterns of rate variation. Proceedings of the National Academy of Sciences of the United States of America 94:7367-7372

Fay MF, Swensen SM, Chase MW (1997) Taxonomic affinities of *Medusagyne oppositifolia* (Medusagynaceae). Kew Bulletin 52:111-120 doi:10.2307/4117844

Gruenstaeudl M, Urtubey E, Jansen RK, Samuel R, Barfuss MHJ, Stuessy TF (2009) Phylogeny of Barnadesioideae (Asteraceae) inferred from DNA sequence data and morphology. Molecular Phylogenetics and Evolution 51:572-587 doi:<http://dx.doi.org/10.1016/j.ympev.2009.01.023>

Hollingsworth ML et al. (2009) Selecting barcoding loci for plants: evaluation of seven candidate loci with species-level sampling in three divergent groups of land plants. Mol Ecol Resour 9:439-457 doi:10.1111/j.1755-0998.2008.02439.x

Oxelman B, Lid, xe, n M, Berglund D (1997) Chloroplast rps16 intron phylogeny of the tribe Sileneae (Caryophyllaceae). Plant Syst Evol 206:393-410

Sang T, Crawford D, Stuessy T (1997) Chloroplast DNA phylogeny, reticulate evolution, and biogeography of Paeonia (Paeoniaceae). American Journal of Botany 84:1120

Taberlet P, Gielly L, Pautou G, Bouvet J (1991) Universal primers for amplification of three non-coding regions of chloroplast DNA. Plant Mol Biol 17:1105-1109 doi:10.1007/bf00037152

Tate JA, Simpson BB (2003) Paraphyly of *Tarasa* (Malvaceae) and diverse origins of the polyploid species. Syst Bot 28:723-737 doi:10.1043/02-64.1

Young ND, Steiner KE, dePamphilis CW (1999) The evolution of parasitism in Scrophulariaceae/Orobanchaceae: Plastid gene sequences refute an evolutionary transition series. Ann MO Bot Gard 86:876-893
